# Supplementary material for: CRISPRoff enables spatio-temporal control of CRISPR editing
Source: Nat Commun. 2020 Oct 7;11:5041. doi: 10.1038/s41467-020-18853-3 (PMC7542431; doi:10.1038/s41467-020-18853-3)
Supplement: Supplementary file 1 — Supplementary Information [file 41467_2020_18853_MOESM1_ESM.pdf]

Supplemental information for:

CRISPRoff enables spatio-temporal control of CRISPR editing

Carlson-Stevermer et. al

SUPPLEMENTAL FIGURES

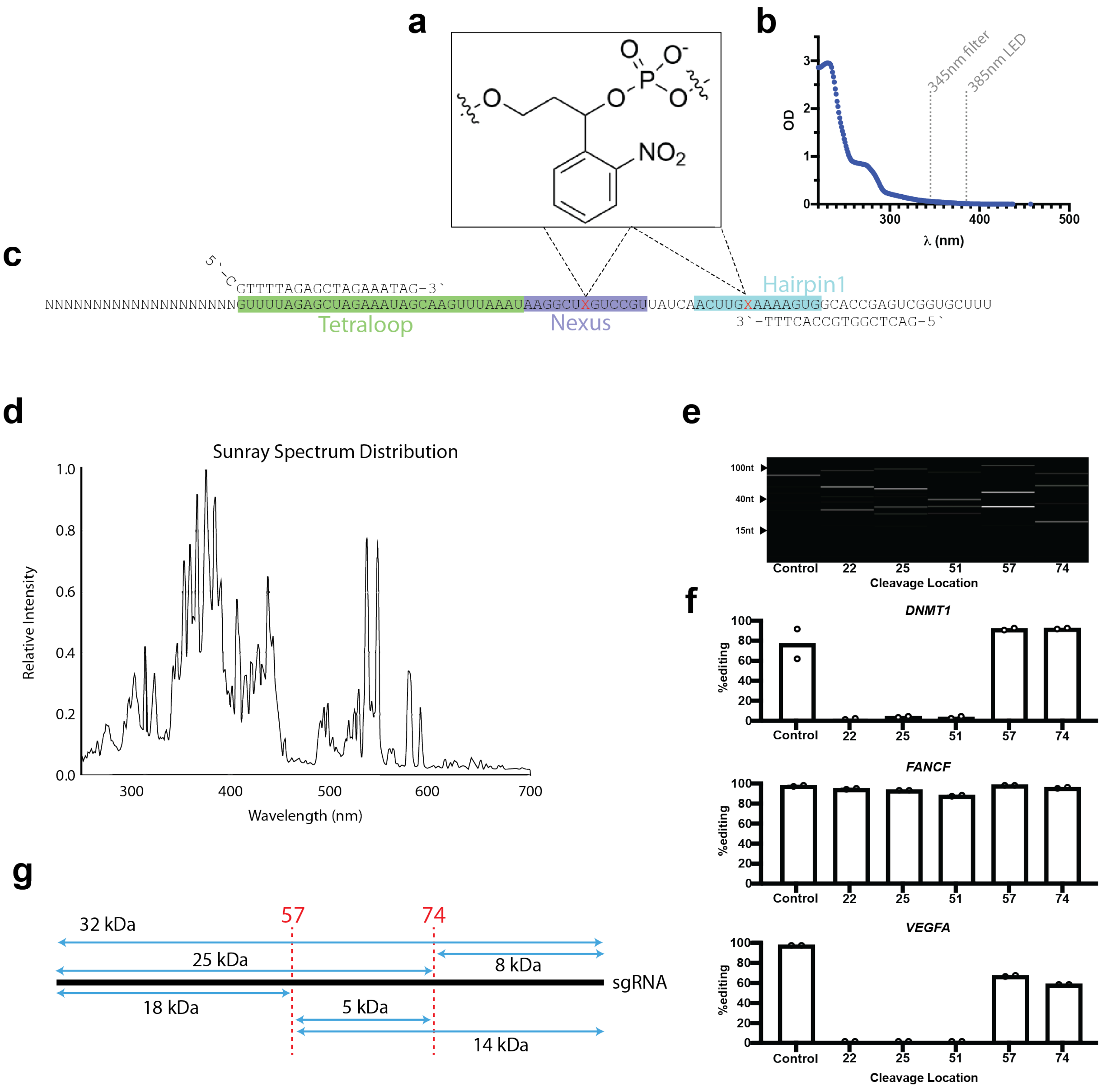

Supplementary Figure 1: a.) Chemical structure of *o*-nitrobenzyl photocleavable (PC) linker incorporated into backbone of CRISPRoff sgRNAS. **b.)** Absorption spectrum of PC linker phosphoramidite solution in acetonitrile. Upon absorbing light at indicated wavelengths, PC linker undergoes selective bond cleavage, resulting in fragmentation of the DBsgRNA backbone. Dotted lines indicate the major wavelengths of light sources used in this work. **c.)** Sequence of CRISPRoff sgRNA backbone, including incorporated

PC linkers (red ‘X’). *Top and Bottom:* overlay of PCR primers used for amplification of sgRNA backbone for ddPCR quantification. **d.)** Spectral output of Sunray UV curing system, the main light source used in this work. **e.)** Fragment analysis of cleaved CRISPRoff sgRNAs with a single replacement nucleotide. All lanes corresponding to CRISPRoff sgRNA show a decrease in full-length RNA and exhibit two bands corresponding to the length of each RNA fragment split at the indicated nucleotide. In these assays some full length sgRNA remained. **f.)** Editing activity of various CRISPRoff spacer locations targeting three genomic loci (*DNMT1*, *FANCF*, and *VEGFA*). Replacing nucleotides with a photocleavable linker in some positions (22) abolished editing efficiency, while others (57, 74) preserved activity (n=2 experimental replicates, data is presented as mean). **g.)** CRISPRoff sgRNA fragments following cleavage. CRISPRoff sgRNAs can be cleaved at either or both locations and rendered inactive. CRISPRoff sgRNAs cleaved at position 57 give rise to fragments of 18 kDa and 14 kDa. CRISPRoff sgRNAs cleaved only at position 74 give rise to fragments of 24 kDa and 8 kDa. CRISPRoff sgRNAs cleaved at both locations give rise to fragments of 18 kDa, 5 kDa, and 8 kDa. All of these species are seen in ESI traces (Figure 1b). Source data are provided as a Source Data file.

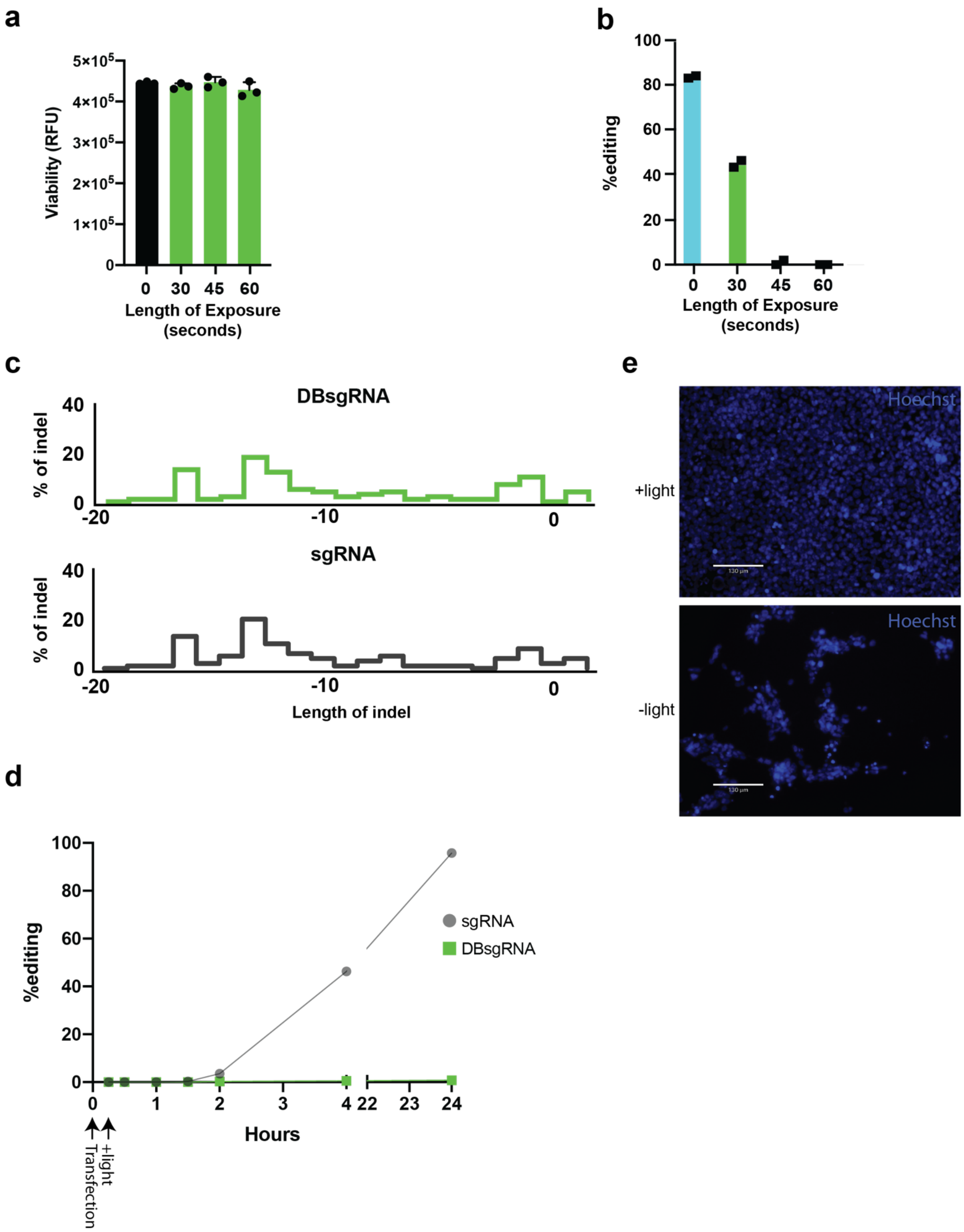
a
Viability (RFU)
5×10^5
4×10^5
3×10^5
2×10^5
1×10^5
0
0 30 45 60
Length of Exposure (seconds)
b
%editing
100
80
60
40
20
0
0 30 45 60
Length of Exposure (seconds)
c
DBsgRNA
% of indel
40
20
0
-20
-10
0
sgRNA
% of indel
Length of indel
e
+light
Hoechst
-light
Hoechst
d
%editing
sgRNA
DBsgRNA
0 1 2 3 4 22 23 24
Hours
Transfection
+light

Supplementary Figure 2: a.) Effect of UV light from curing lamp on cell viability. Cells were left in the dark or exposed to light under a 345 nm longpass filter. This filter removes all wavelengths shorter than 345 nm that may cause DNA damage. Using these filters, cell viability was not significantly different compared to unexposed cells (n=3 technical replicates *p=0.27*, one-way ANOVA, data is presented as mean±1 SD). **b.)** Effect of light exposure duration on ablation of editing. Longer periods of exposure were more efficient at cleaving DBsgRNAs, achieving complete ablation between 45-60 seconds (n=2 biological replicates, data is presented as mean). **c.)** Indel profile of CAMK1 DBsgRNA and standard sgRNA. Indel profiles of both sgRNAs are similar, suggesting that DBsgRNAs do not change the repair mechanism of DSBs. **d.)** High resolution analysis of editing time course at the *FANCF* locus. Transfected cells were exposed to light 15 minutes post-transfection. Genomic DNA was harvested 15, 30, 60, 90, 120 minutes and 4 and 24 hours post-transfection. Using standard sgRNAs, 45% of alleles had already undergone a DSB and been repaired with an indel mutation 4 hours post-transfection. This activity was not seen using CRISPRoff sgRNAs suggesting editing at this locus can be controlled. **e.)** Editing using a DBsgRNA with an off-target site in a known essential gene. Lack of inactivation caused high degree in cell death as seen through loss of confluency. Inactivation of DBsgRNA rescued viability in these cells (image representative of 3 independent experiments, scale bar: 130 µm). Source data are provided as a Source Data file.

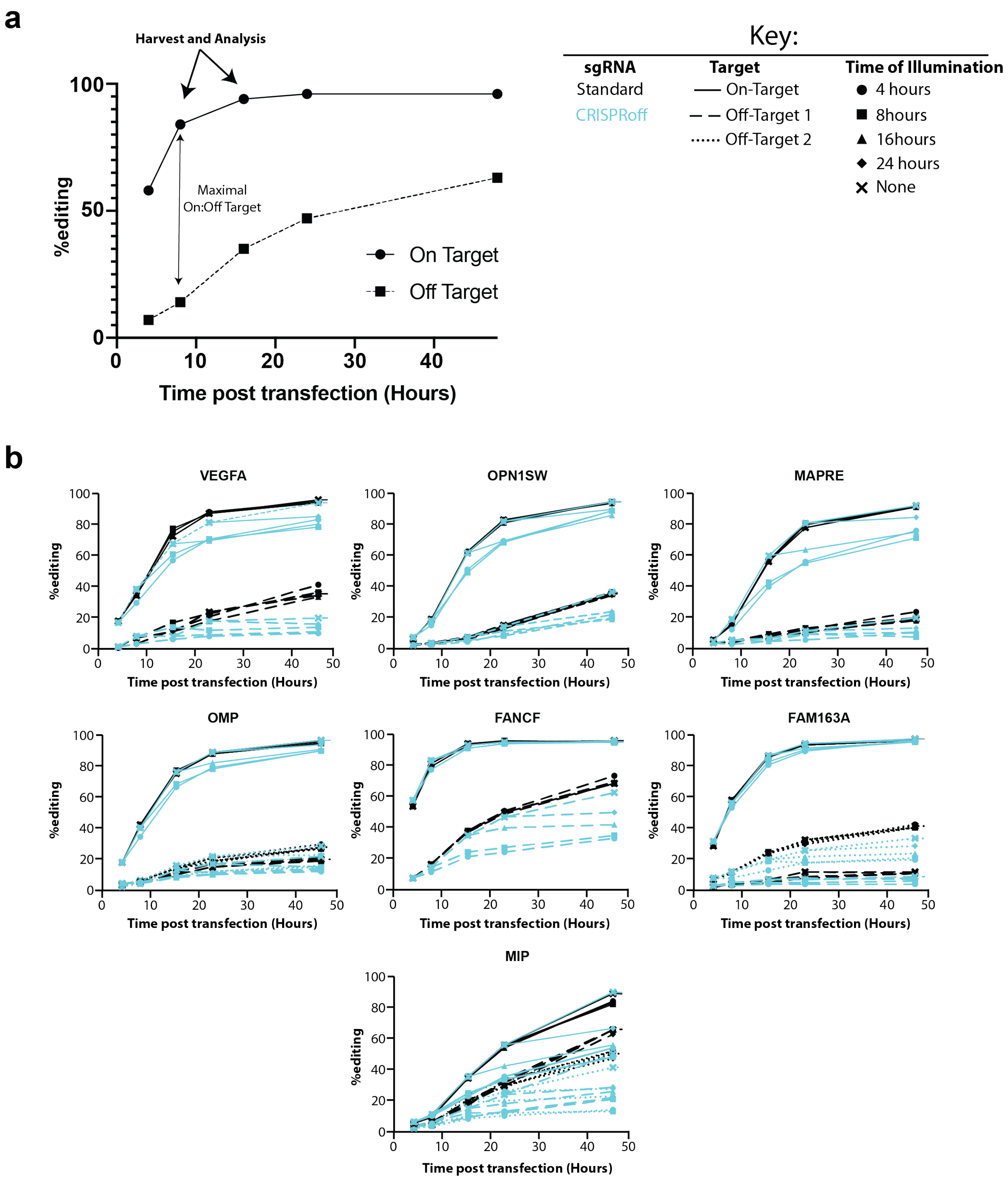

Supplementary Figure 3: **a.)** Ratio of on:off-target editing at various time points post transfection at the *FANCF* locus. On-target gene editing happens quickly and reaches saturation while off-target editing lags before becoming saturated at later timepoints. Identification of these inflection points can minimize the harm of off-target editing events.

b.) Absolute values of on and off-target editing events at seven genomic loci. Cells were transfected with either standard sgRNAs or CRISPRoff sgRNAs. Cell pools were then illuminated at either 4, 8, 16, 24, or 48 hours post transfection. At these same time points, separate cell pools were harvested and analyzed for the presence of indels. Off-target effects were lower at assay endpoint in samples that were illuminated sooner after transfection. Source data are provided as a Source Data file.

SUPPLEMENTARY TABLES

Supplementary Table 1: List of sgRNAs used

Target	Protospacer Sequence
AAVS1	GGGGCCACTAGGGACAGGAT
BUB1B	AGTGAAGCCATGTCCCTGGA
CAMK1_sg1	TGCCAGGATCACCTCCGAGA
CAMK1_sg2	GCGTCCTCTTATCTTCTGCC
CEL	AACCAGTTGCAGGCGCCCCA
Chr8q23_sg1	TTATAGTTACGATGTTTGAT
Chr8q23_sg2	AGTCTACTATGAGTTTTCTG
CXCR4	GATAACTACACCGAGGAAAT
DNMT1	GGAGTGAGGGAAACGGCCCC
EMX1	GAGTCCGAGCAGAAGAAGAA
FAM163A	CTGCAGGGCTCGCTGGTGAG
FANCF	GCTGCAGAAGGGATTCCATG
GAA	AGGAGCCGGTGGGAGCAGGG
GRK1	GCCGTCAAAGCTGCCTCGGG
ITGA7	GGTGCTGGAGGGCGAGGCTG
IRAK4	GTCCTGTCTTTGTCACAGAA
MAPRE1	TTCTCTGCAGATAATTCCTG
MIP	GCTGGGGTCCTCACTGCGCT
OMP	GAACTGTAGCCGCTGCTGCT
OPN1SW	ACAGGGGCAATGTGGTACTG
PRGN	CAGATGCCTGCTCAGTGTTG
PRKAG3_sg1	AGCAAGAAAACAGCAGCTCA
STK3_sg1	AAAGCAATACACAAGGAATC
STK3_sg2	CCATAATGCAGCAATGTGAC
VEGFA	GGTGAGTGAGTGTGTGCGTG
GFP-C1	CTTCAGGGTCAGCTTGCCGT

Supplementary Table 2: List of primers used

Target	Primer F	Primer R	Primer Seq
AAVS1	GCCCCTATGTCCACTTCAGG	CTCAGGTTCTGGGAGAGGGT	CTCCATCGTAAGCAAACCTTAGAGG
BUB1B	AGAAATCCTCCCACTTCGGC	GCAGATTCTTGTGCCAGTGC	CAGCTAACAAAGAAGCTTAGGCATATAATA
CAMK1_sg1	ACAACCCTGCCAAGTGGAAA	ACTAGGGGAGGGTCATCCAC	CATTTTATAAAGGGGCAATTTAAGGCTTAG
CAMK1_sg2	ACAACCCTGCCAAGTGGAAA	ACTAGGGGAGGGTCATCCAC	CATTTTATAAAGGGGCAATTTAAGGCTTAG
CEL	CTGAGGGTGTAGAGGGGAGG	GTTCTACCTGGCACCTGTCC	CCTGAGAGCTCATGAACAAGCAT
Chr8q23_sg1	CTCGTCAAAACAAGGGTAAGCA	GTTTGAGTTGACCAAACGCA	CAAGGGTAAGCAAAGAAATAAAATCTCTTC
Chr8q23_sg2	ACCTGTCACATTGCTGCATT	GTTTGAGTTGACCAAACGCA	TTGATTATTTCCTGAAGATCTGATTCAACA
CXCR4	TTGTGCCCTTAGCCCACTAC	CCAGAAGGGAAGCGTGATGA	GTACTTGTCCGTCATGCTTCTCAGTTT
DNMT1	GATCAAGCTTTGTATGTTGGCCAA	AATCCAGAATGCACAAAGTACTGC	GATCAAGCTTTGTATGTTGGCCAA
EMX1	CAGCTCTGTGACCCTTTGTTTG	ACTAAACTACAGTGGTGCCTGG	CAGCTCTGTGACCCTTTGTTTG
FAM163A	GAGTGGTGGGAGGGGAAAAG	CATGTCAGCCGTCCGTATGT	CTTGCAAAGCTGGGATTAGAAACTT
FANCF	GATATTTCCAAAGCGAAAGGAAGC	ATCAGAGAGTCCTCCTGGAGATTT	GATATTTCCAAAGCGAAAGGAAGC
GAA	GGTGAGTCTCCTCCAGGACT	CAGACTGTGCAAGTGCTCTG	CTTTTCTCGCCCTTCCTTCTGG
GRK1	GTCTCTCTCGTCCAGCAAGGG	ATGTCTTTCCAGAGCTCCAGGG	GTCTCTCTCGTCCAGCAAGGG
ITGA7	GGTTGTCGCCAAACCTTCAC	GGGATTGGGGAGTCAAGAGC	GAGTCAAGAGCACAAGAAACATGAGAACAT
IRAK4	GCTTCTTGTGTGTGCTGTGAG	GCCTGTGATTGCTGCACAAA	CAAGTTTCTAGTTTAACTTTTTCACAACCA
MAPRE1	GGTACTCTTGAAGGCAAACTGC	CGCTGAATGAATATCTGGAACGC	ACTGCATGAAACTTGCTTTATAAATTTAGG
MIP	TCAGCCAACCATTACCGTGT	TAAAGGGGACTGTCCACCCA	CATTACCGTGTTGAGTGCTAGGTTTC
OMP	TTGAGAACTGAGTGGGGCTG	GCGTGTCATGAGGTTGGTGA	TTGAGAACTGAGTGGGGCTG
OPN1SW	CCCCTAACCCCTTTTTCCCC	GTTTTGTGGGGTGGGAGGAT	CTAACCCCTTTTTCCCCTGCAGTAC
PRGN	TGAGCTGGGTGGCCTTAACA	CATTGGCAGGGCCCTTTTATC	CCAGATGGTCAGTTCTGCCC
PRKAG3_sg1	ATGTAGGGAGACTGAGGCCA	GCCCATTGGAAGCTTGCAAA	TTGGGTCCAACTCTGTGTTATGGAG
STK3_sg1	ACGGCAAAACCCTGTCTCAA	TCCACAGAAAACTCATAGTAGACTT	AAACAAGGGTAAGCAAAGAAATAAAATCTC

STK3_sg2	AAGCCATCCTCATCTGCCTT	ACACAAGGAATCCGGTCAAGT	GGAGAAACCCATCTCTACTAAAAATACAAA
VEGFA	GAAGCAACTCCAGTCCCAAATATG	GTTCACAGCCTGAAAATTACCCAT	GAAGCAACTCCAGTCCCAAATATG

Supplementary Table 3: ddPCR primers

Primer Name	Sequence
sgRNA_F	CGTTTTAGAGCTAGAAATAGC
sgRNA_R	GACTCGGTGCCACTTT

Supplementary Table 4: List of off-target sites

Target	On Target Sequence	Off Target Sequence
MIP_OT1	GCTGGGGTCCTCACTGCGCT	AGTGGGGTCCTCACTGCACT
MIP_OT2	GCTGGGGTCCTCACTGCGCT	TGTGGGGCACTCACTGCGCT
FAM163_OT1	CTGCAGGGCTCGCTGGTGAG	CTGCAGGGCCCGCTGGAGAG
FAM163_OT2	CTGCAGGGCTCGCTGGTGAG	CTGCAGGGGACACTGGTGAG
OMP_OT1	GAACTGTAGCCGCTGCTGCT	AGGCTGTAGCCCCTGCTGCT
OMP_OT2	GAACTGTAGCCGCTGCTGCT	GAACTACAGCCACTGCTGCT
FANCF_OT1	GCTGCAGAAGGGATTCCATG	GCTGCAGAAGGGATTCCAAG
MAPRE_OT1	TTCTCTGCAGATAATTCCTG	ATCTCTGCAGATAATCCCTG
OPN1SW_OT1	ACAGGGGCAATGTGGTACTG	TTAGAGGCAATGTGGTACTG
VEGFA_OT1	GGTGAGTGAGTGTGTGCGTG	TGTGGGTGAGTGTGTGCGTG

Supplementary Table 5: List of off-target sequencing primers

Target	Primer F	Primer R	Primer Seq
MIP_OT1	CTCACAGCAAGGTCGACCAC	CACCCCTACACACTGCCTTT	CATTCGAAATCCTATGCTGAGCTTTCATAG
MIP_OT2	CGGCTCCAGTGCTCTTTCTT	GGAGGGTACGCAAGGTTTGG	GCCTTTCTGACTCCCATCCTTC
FAM163_OT1	GTGGATAGGAGCATCTGCCC	GTGGGAGAAGGAGGTCATGC	CCTCCCCATATGCTTGGAGTAAG
FAM163_OT2	GCCCACATTTGCACTGACTC	GATCATGGTGATGTGCGCAC	AGACAAGACACCACAGCAATTCCAATTTTG
OMP_OT1	AGATCCTGGGGGTCTCTGTG	CGCCTGCTTATCATTTGGGC	GAACTAGAGACTTATGAGTGGTTCTAAGAT
OMP_OT2	TTGCAACACCAGGGCTTTCT	CTTCACAGGCTTCAGGGAGG	TAGCATTTCCTTCTTTAGAGGTTGATTATG
FANCF_OT1	AGTTTCACATCCCTGTCTTACCTC	AGACTCACAACATCCATCAGAACA	AGTTTCACATCCCTGTCTTACCTC
MAPRE_OT1	ACAGTTTGTGGGCTTTTTGGT	GCATTCTGCCCTGTTTGTGG	CATTTTGAGCAAGGTCAGAAGGAC
OPN1SW_OT1	TGGCCATAGGAAGCACAGTC	ATGATCCCCCTGTCTCTGCT	CTACCTCCCTCTCCTTAGCTTCTC
VEGFA_OT1	AGGGACTTGAGTATCTGCAGTTTT	TGAAGAGATATCTGCACCCTCATG	AGGGACTTGAGTATCTGCAGTTTT
