# Supplementary material for: CRISPRoff enables spatio-temporal control of CRISPR editing
Source: Nat Commun. 2020 Oct 7;11:5041. doi: 10.1038/s41467-020-18853-3 (PMC7542431; doi:10.1038/s41467-020-18853-3)
Supplement: Supplementary file 3 — Reporting Summary [file 41467_2020_18853_MOESM3_ESM.pdf]

nature research

Corresponding author(s): Carlson-Stevermer, Jared

Last updated by author(s): 2020-8-31

Reporting Summary

Nature Research wishes to improve the reproducibility of the work that we publish. This form provides structure for consistency and transparency in reporting. For further information on Nature Research policies, see our Editorial Policies and the Editorial Policy Checklist.

Statistics

For all statistical analyses, confirm that the following items are present in the figure legend, table legend, main text, or Methods section.

n/a	Confirmed	
☐	☒	The exact sample size (n) for each experimental group/condition, given as a discrete number and unit of measurement
☐	☒	A statement on whether measurements were taken from distinct samples or whether the same sample was measured repeatedly
☐	☒	The statistical test(s) used AND whether they are one- or two-sided<br>*Only common tests should be described solely by name; describe more complex techniques in the Methods section.*
☒	☐	A description of all covariates tested
☐	☒	A description of any assumptions or corrections, such as tests of normality and adjustment for multiple comparisons
☐	☒	A full description of the statistical parameters including central tendency (e.g. means) or other basic estimates (e.g. regression coefficient) AND variation (e.g. standard deviation) or associated estimates of uncertainty (e.g. confidence intervals)
☐	☒	For null hypothesis testing, the test statistic (e.g. F, t, r) with confidence intervals, effect sizes, degrees of freedom and P value noted<br>*Give P values as exact values whenever suitable.*
☒	☐	For Bayesian analysis, information on the choice of priors and Markov chain Monte Carlo settings
☒	☐	For hierarchical and complex designs, identification of the appropriate level for tests and full reporting of outcomes
☒	☐	Estimates of effect sizes (e.g. Cohen's d, Pearson's r), indicating how they were calculated

Our web collection on statistics for biologists contains articles on many of the points above.

Software and code

Policy information about availability of computer code

Data collection	ddPCR data was collected with QuantaSoft v1.7
Data analysis	Sanger sequencing was analyzed using ICE v2. ddPCR data was analyzed with QuantaSoft V 1.7. All data was analyzed and presented using Prism 8.

For manuscripts utilizing custom algorithms or software that are central to the research but not yet described in published literature, software must be made available to editors and reviewers. We strongly encourage code deposition in a community repository (e.g. GitHub). See the Nature Research guidelines for submitting code & software for further information.

Data

Policy information about availability of data

All manuscripts must include a data availability statement. This statement should provide the following information, where applicable:
- Accession codes, unique identifiers, or web links for publicly available datasets
- A list of figures that have associated raw data
- A description of any restrictions on data availability

All data is available upon request. Raw Sanger traces are also available at zenodo accession 4009446 http://doi.org/10.5281/zenodo.4009446

Field-specific reporting

Life sciences study design

All studies must disclose on these points even when the disclosure is negative.

Sample size	No sample size calculation was performed. Sample size was chosen to be sufficient to observe distinctions between treatments across multiple target sites. Sample sizes and statistical data are reported in associated figure legend.
Data exclusions	All data that was successfully analyzed was included.
Replication	Finding were replicated by independently repeating experiments in different cell lines. Each experiment was conducted with a independent synthesis of sgRNA. All experiments were separated by at least one week and findings were replicated in all attempts.
Randomization	Cell pools were split into plus/minus treatment groups at random and analyzed independently alongside controls.
Blinding	Analysis was automated using a batch algorithm to ensure all data was equivalently processed. No human/animal subjects were used that would bias group analysis

Reporting for specific materials, systems and methods

We require information from authors about some types of materials, experimental systems and methods used in many studies. Here, indicate whether each material, system or method listed is relevant to your study. If you are not sure if a list item applies to your research, read the appropriate section before selecting a response.

Materials & experimental systems

n/a	Involved in the study	
☒	☐	Antibodies
☐	☒	Eukaryotic cell lines
☒	☐	Palaeontology and archaeology
☒	☐	Animals and other organisms
☒	☐	Human research participants
☒	☐	Clinical data
☒	☐	Dual use research of concern

Methods

n/a	Involved in the study	
☒	☐	ChIP-seq
☒	☐	Flow cytometry
☒	☐	MRI-based neuroimaging

Eukaryotic cell lines

Policy information about cell lines

Cell line source(s)	HEK293, U2OS, and Hep3B cell lines were all obtained from ATCC
Authentication	No cell lines underwent secondary identity confirmation
Mycoplasma contamination	Cell lines underwent monthly tests for mycoplasma. No positive tests were observed.
Commonly misidentified lines (See ICLAC register)	HEK293 cells were used as they are common in many laboratories, demonstration of technology in this line enables replication by other researchers.
